# Sinomenine ameliorates adjuvant-induced arthritis by inhibiting the autophagy/NETosis/inflammation axis

**DOI:** 10.1038/s41598-023-30922-3

**Published:** 2023-03-09

**Authors:** H. Jiang, Q. Lu, J. Xu, G. Huo, Y. Cai, S. Geng, H. Xu, J. Zhang, H. Li, K. Yuan, G. Huang

**Affiliations:** 1 School of Life Sciences, Beijing University of Chinese Medicine, Beijing, People’s Republic of China; 2 School of Chinese Materia, Beijing University of Chinese Medicine, Beijing, People’s Republic of China; 3 Dongfang Hospital, Beijing University of Chinese Medicine, Beijing, People’s Republic of China; 4 School of Clinical Traditional Chinese Medicine, Capital Medical University, Beijing, People’s Republic of China

**Keywords:** Autoimmunity, Chronic inflammation

## Abstract

Studies have found that neutrophil extracellular traps (NETs) which are the specific dying form of neutrophil upon activation have fundamental role in the rheumatoid arthritis onset and progression. The purpose of this study was to explore the therapeutic effect of Sinomenine on adjuvant-induced arthritis in mice, and the neutrophil activities regulated by Sinomenine. The rheumatoid arthritis model was established by local injection of adjuvant and the Sinomenine treatment was administered orally for 30 days, during which, arthritic scores were evaluated and the joint diameter was measured to determine disease progression. The joint tissues and serum were acquired for further tests after sacrifice. Cytometric beads assay was performed to measure the concentration of cytokines. For paraffin-embedded ankle tissues, hematoxylin and erosin staining and Safranin O-fast staining were adopted to monitor the tissue changes of joint. In order to analyze the inflammation, NETs and autophagy of neutrophils in vivo, immunohistochemistry assays were applied to detect the protein expression levels in the local joints. To describe the effect brought by Sinomenine on inflammation, autophagy and NETs in vitro, the western blotting and the immunofluorescence assays were performed. The joint symptoms of the adjuvant induced arthritis were alleviated by the Sinomenine treatment significantly in terms of the ankle diameter and scores. The improvement of local histopathology changes and decrease of inflammatory cytokines in the serum also confirmed the efficacy. The expression levels of interleukin-6, P65 and p-P65 in the ankle areas of mice were remarkably reduced by Sinomenine. Compared with the model group, the decreased expression levels of lymphocyte antigen 6 complex and myeloperoxidase in the Sinomenine treating group showed the inhibitory effect of Sinomenine on the neutrophil migration. The expression of protein arginine deiminase type 4 (PAD4), ctrullinated histone H3 (CitH3) and microtubule-associated protein 1 light chain 3B (LC3B) had the similar tendency. Upon activation of lipopolysaccharide (LPS) in vitro, Sinomenine suppressed the phosphorylation of P65, extracellular signal-regulated kinase (ERK) and P38 of neutrophil. Meanwhile, Sinomenine inhibited NETs formation induced by phorbol 12-myristate 13-acetate (PMA), which were demonstrated by the decreased expression of neutrophil elastase (NE), PAD4 and CitH3. Sinomenine also inhibited PMA-induced autophagy in vitro based on the changes of Beclin-1 and LC3B. Sinomenine has good efficacy in treating adjuvant induced arthritis via regulating neutrophil activities. Apart from inhibiting activation of nuclear factor kappa-B (NF-κB) and mitogen-activated protein kinase (MAPK) pathways, the mechanism includes suppression of NETs formation via autophagy inhibition.

## Introduction

Rheumatoid arthritis (RA) has become a global health issue^1^. The global prevalence of RA is approximately 1%. Women are more vulnerable than men^2,3^. It affects an estimated 1.5 million patients in the United States^4^ and the annual cost of arthritis, including osteoarthritis and rheumatoid arthritis, exceeds $200 billion^5,6^.

The clinical manifestations of RA vary greatly among individuals. The main joint discomforts include morning stiffness, joint swelling and joint deformities. Extra-articular symptoms include rheumatoid nodules in the skin, rheumatoid vasculitis, pericarditis, pleurisy and so on^7^. Its insidious onset is correlated with its complicated etiology. The genetic factors^8,9^ and environmental factors both contribute to the onset of RA^10^. The epigenetic modifications brought by environmental changes^11^ have been considered susceptible factors^12^. In addition, the intestinal microbiota affects the developmental progression of RA. Alterations in the oral and gut microbiota are related to increasing levels of C-reactive protein (CRP) and the emergence of antibodies to citrullinated protein antigens (ACPAs)^13^.

Neutrophils are the fundamental immune cells in the host. Upon activated in the early stage of RA, neutrophils are migrating to the joint cavity and secreting chemokines and cytokines. In addition, they involve in the inflammatory state in the form of neutrophil extracellular traps (NETs). The process of NETs formation is called NETosis, during which nucleic acid fibers and bactericidal proteins are released^14^. In NETs, degradation of the actin cytoskeleton is initiated by myeloperoxidase (MPO). Activated neutrophil elastase (NE) and protein arginine deiminase type 4 (PAD4) participate in chromatin destruction^15^. PAD4 is a calcium-dependent enzyme that catalyzes the transformation from arginine to citrulline as a posttranslational protein modification^16^. Citrullinated histones promote nucleic acid depolymerization^17^ and are likely to be recognized as autoantigens, ultimately stimulating the production of antibodies. The intensified responses greatly exacerbate RA symptoms^18^. Therefore, we propose that neutrophils may be an important target for the treatment of RA.

Sinomenine (9α,13α,14α-7,8-didehydro-4-hydroxy-3,7-dimethoxy-17-methylmorphane-6-one hydrochloride) has extensive pharmacological activities. Sinomenine can be used to treat cancer^19–23^, protect the cardiovascular system^24^, relieve chronic inflammatory diseases^25^, and alleviate acute injury to the lung^26^ and liver^27^. Recent studies have revealed that Sinomenine is effective in the treatment of RA with lower toxicity and fewer side effects. Weiwei Liu et al. found that Sinomenine could clinically ameliorate the disease activity of RA patients and reduce their percentage of peripheral monocytes^28^. In a randomized controlled clinical trial, Run-yue Huang et al. found that compared with RA patients treated with methotrexate and leflunomide (MTX + LEF), patients treated with methotrexate and Sinomenine (MTX + SIN) gained similar medical benefits with fewer adverse events^29^. Experimental studies have revealed a possible mechanism by which Sinomenine regulates RA. Yue Sun et al. found that the combination of Sinomenine and methotrexate (MTX + SIN) could significantly inhibit the activation of synovial receptor activator of NF-κB ligand (RANKL) and osteopontin (OPN) production in collagen-induced arthritis (CIA) rats^30^. Zhi-tao Feng et al. found that in CIA mice, hypoxia-inducible factor-1α (HIF-1α), vascular endothelial growth factor (VEGF) and angiopoietin 1 (ANG-1) were regulated by Sinomenine to decrease angiogenesis^31^. In addition, a new drug delivery system loaded with Sinomenine exhibited superior anti-rheumatoid arthritis effects in vivo and in vitro^32,33^.

In this article, we established a mouse model of adjuvant-induced arthritis (AA) to determine the therapeutic effect of Sinomenine on rheumatoid arthritis and to explore its specific mechanism, focusing on neutrophil activities.

## Materials and methods

### Animals

C57BL/6 mice (female, 7–8 weeks old) were purchased from Beijing Vital River Laboratory Animal Technology Co., Ltd. All experimental procedures were reviewed and approved by the Animal Care and Use Committee of the Beijing University of Chinese Medicine. All the experiments were performed and analyzed in accordance with ARRIVE guidelines. All the following methods were performed in accordance with the relevant guidelines and regulations.

### Drugs and chemicals

Freund’s complete adjuvant (FCA) (F5881), lipopolysaccharide (LPS) (L2880) and phorbol 12-myristate 13-acetate (PMA) (P1585) were obtained from Sigma Chemicals (Louis, MO, USA). Sinomenine (S2359) was purchased from Selleck Chemicals (Shanghai, CHN). Anti-MPO (ab9535), anti-NE (ab21595), anti-Ly6G (ab25377), anti-CitH3 (ab5103), anti-IL-6 (ab208113), anti-LC3B (ab48394), anti-Beclin-1 (ab207612), anti-GAPDH (ab181602), anti-ERK1/2 (ab115799), anti-rabbit IgG secondary (ab6721) and goat anti-rabbit IgG H&L (Alexa Fluor® 555) secondary antibodies (ab150086) were obtained from Abcam (Cambridge, MA, USA). The anti-p65 (3033S), anti-phospho-p65 (Ser536) (6956S), anti-SAPK/JNK (9252S), anti-phospho-SAPK/JNK (Thr183/Tyr185) (9251S), anti-phospho-ERK1/2 (Thr202/Tyr204) (9101S), anti-p38 (9212S) and anti-phospho-p38 (Thr180/Tyr182) (9211S) antibodies were purchased from Cell Signaling Technology (Danvers, MA, USA). The anti-PAD4 antibodies (17373-1-AP) were purchased from Proteintech Group, Inc. (Wuhan, HB, CHN). Horseradish peroxidase (HRP)-conjugated goat anti-mouse/rabbit IgG polymer kit was purchased from ZS GB-Bio (Beijing, CHN). Percoll™ PLUS (17-5445-01) was purchased from GE Healthcare (Uppsala, Sweden). The Cytometric Beads Array (CBA) kit (560485) was purchased from BD Biosciences (Becton, Dickinson and Company). Tris-buffered saline Tween-20 (TBST) was purchased from Biorigin (Beijing, CHN). Sodium citrate antigen retrieval solution and RIPA buffer were obtained from Solarbio (Beijing, CHN). The enhanced chemiluminescence (ECL) reagent was obtained from Cwbio IT Group (Beijing, CHN). Cell Counting Kit 8 (CCK-8) was purchased from Analysis Quiz (Beijing, CHN).

### Induction of adjuvant-induced arthritis

C57BL/6 mice (female, 7–8 weeks old) were used to establish the AA model. After general anesthesia, injection of 100 μL vortexed FCA was conducted with 1 mL insulin syringes following these steps: first, 20 μL was injected into the ankle joint cavity of the left hind foot, and then the remaining 80 μL was injected in four doses into the tissues around the joint. Three days after injection, the left hind ankle joint of the mouse was scored: 0-normal, 1-slight redness or swelling of the ankle joint, 2-moderate swelling and slight limitation of movement, 3-obvious swelling and limitation of movement, 4-severe swelling and movement disorders. The joint diameter was measured with a pocket thickness meter every 3 days. At the same time, 90 mg/kg/d Sinomenine was given to the treatment group by intragastric administration starting three days after FCA injection and continuing for 30 consecutive days. The control mice and AA model mice received normal saline.

### Specimen collection

On Day 33, the mice were anaesthetized with isoflurane, and anesthesia of each mouse was maintained in a single breathing unit. The blood was collected by cardiac puncture. The mice were sacrificed with CO_2_ in a gas chamber (Yuyan, Shanghai, China). The collected blood was placed at room temperature for 2 h and then centrifuged for 30 min at 3000 rpm to obtain 200–300 μL serum. At the same time, the left hind limbs of mice with representative joint diameters and arthritis scores from each group were collected and fixed for further experiments.

### Histopathology and immunohistochemistry

After fixation in 4% paraformaldehyde, the joint tissues were decalcified with 10% EDTA solution (pH 7.2) for 1–2 months. After embedding in paraffin, the tissues were sliced into 4 μm sections. H&E and safranin O-fast green staining were performed according to standard procedures. In short, for the H&E staining, the sample slides were dewaxed with xylene and ethanol and then stained with hematoxylin and eosin after hydration. Finally, the slides were dehydrated with ethanol and xylene. For safranin O-fast green staining, after routine dewaxing and hydration steps, hematoxylin, fast green and safranin O were applied successively. After dehydration, the slides were sealed and examined under a microscope. The percentage of the cartilage area was quantified by ImageJ. We randomly selected three areas in the image for statistical analysis. For IHC, standard procedures were applied. In short, after hydration and permeabilization, 3% H_2_O_2_ was used to remove endogenous peroxidase. For permeabilization, 0.1% Triton X-100 was gently dripped onto the tissue section and incubated at 37 °C for 20 min. Sodium citrate antigen retrieval solution was used for antigen retrieval, and 10% goat serum was used for blocking. For antigen retrieval, 1 L of sodium citrate antigen retrieval solution (Solarbio, C1032) was heated to boiling in a pressure cooker. The sections were put into the pressure cooker and heated at high (2100 W) until steam came out of the top, then the pressure cooker was turned down to low heat (800 W) for 2.5 min. Goat serum was then incubated with the slides for blocking, followed by the primary antibody and the secondary antibody. The target proteins were finally developed with 3,3′-diaminobenzidine (DAB). ImageJ software (National Institutes of Health, Bethesda, MD, USA) was used to quantify the IOD of the positive area.

### Detecting inflammatory cytokines in serum using flow cytometric assays

The experimental operations were carried out following the instructions of the Cytometric Beads Array (CBA) kit (Cat: 560485, BD biosciences, USA). First, the standard samples were diluted. Then, 50 μL of a mixture of capture beads was added to the standard tubes and sample tubes and mixed well, followed by 50 μL Th1/Th2/Th17 PE detection reagent being added to each tube. The tubes were incubated in the dark at room temperature for 2 h. After washing, the precipitates were resuspended and detected by the FACS Calibur (LSRFortessa SORP, BD biosciences, USA), followed by analyzation using FCAP Array software (Cat: 652099, BD biosciences, USA).

### Neutrophils preparation and culture

Filtered 1 mL of 10% protease peptone was injected intraperitoneally into the mice on the first day. The second injection was given after 12 h. Four hours later, peritoneal lavage cells were obtained by washing the abdominal cavity with RPMI-1640 supplemented with 10% FBS. After centrifugation, the cell pellets were resuspended and subjected to density gradient centrifugation with Percoll™ PLUS to obtain neutrophils. A flow cytometric assay was performed to detect the purity of the separated neutrophils (Supplement Figure 1). The purity of neutrophils = number of Ly6G-positive cells (neutrophils)/total number of living cells × 100%. After treatment, the cells were collected for Western blot analysis and other tests. In a previously published article, our laboratory specifically introduced the method of separating and extracting mouse peritoneal neutrophils^34^.

### CCK-8 assay

Neutrophils isolated from the peritoneal cells of mice were inoculated into 96-well plates (100 μL/well, 5 × 10^6^ cells/well) with or without different concentrations of Sinomenine. The neutrophils were cultured at 37 °C in a 5% CO_2_ incubator. After four hours of culture, Cell Counting Kit 8 (CCK-8) solution (10 μL/well) was added to each well and incubated for 1 h. The absorbance at 450 nm was measured with a microplate reader (BioTek Instruments, Inc).

### Western blot analysis

Protein samples were processed by RIPA buffer and separated by sodium dodecyl sulfate–polyacrylamide gel electrophoresis (SDS–PAGE, 12%). The proteins were transferred to polyvinylidene fluoride (PVDF) membranes. After blocking with 5% nonfat dry milk or 3% bovine serum albumin (BSA) in TBST buffer for 2 h, the membranes were incubated with primary antibodies overnight at 4 °C. The membranes were incubated with HRP-conjugated secondary antibody for 1 h at room temperature after washing three times with TBST. Proteins were detected by ECL reagent and visualized. ImageJ software was used to quantify the grayscale of the bands.

### Immunofluorescence staining

After counting, the extracted neutrophils were inoculated into a laser confocal dish and cultured at 37 °C in 5% CO_2_ for 4 h. Then, the neutrophils were fixed with 4% paraformaldehyde for 20 min after 3 washes with prechilled phosphate buffered saline (PBS). The cells were immersed in 0.1% Triton X-100 for 20 min at room temperature. Then, the cells were blocked with 3% BSA for 1 h at room temperature. The primary antibody incubation was conducted overnight at 4 °C, followed by incubation with Alexa Fluor-conjugated secondary antibodies in the dark. The nuclei were stained with DAPI at room temperature for 15 min. The fluorescent signals were collected with a confocal microscope (TCS SP8, Leica). ImageJ software was used to measure the staining intensity and nuclear areas. The cell nucleus was circled with the curve tool and its area was measured with standardized scales. Then, further statistical analysis was performed using SPSS.

### Statistical analysis

The results are expressed as the mean ± SD and were calculated by SPSS (IBM SPSS Statistics Software, IBM, NYC, USA). Two-way analyses of variance (ANOVA) were used to analyze the ankle diameters, arthritic scores and body weight of all groups. For the western blot, immunofluorescence and CBA experiments, the statistical significance of differences was determined using one-way ANOVA. Post hoc least significant difference (LSD) test was also applied to compare the statistical significance of differences in immunohistochemical staining of inflammation, autophagy and NETosis. *P* < 0.05 was considered statistically significant.

## Results

### Sinomenine reduced ankle joint swelling and arthritis scores in AA mice

**Figure 1:**
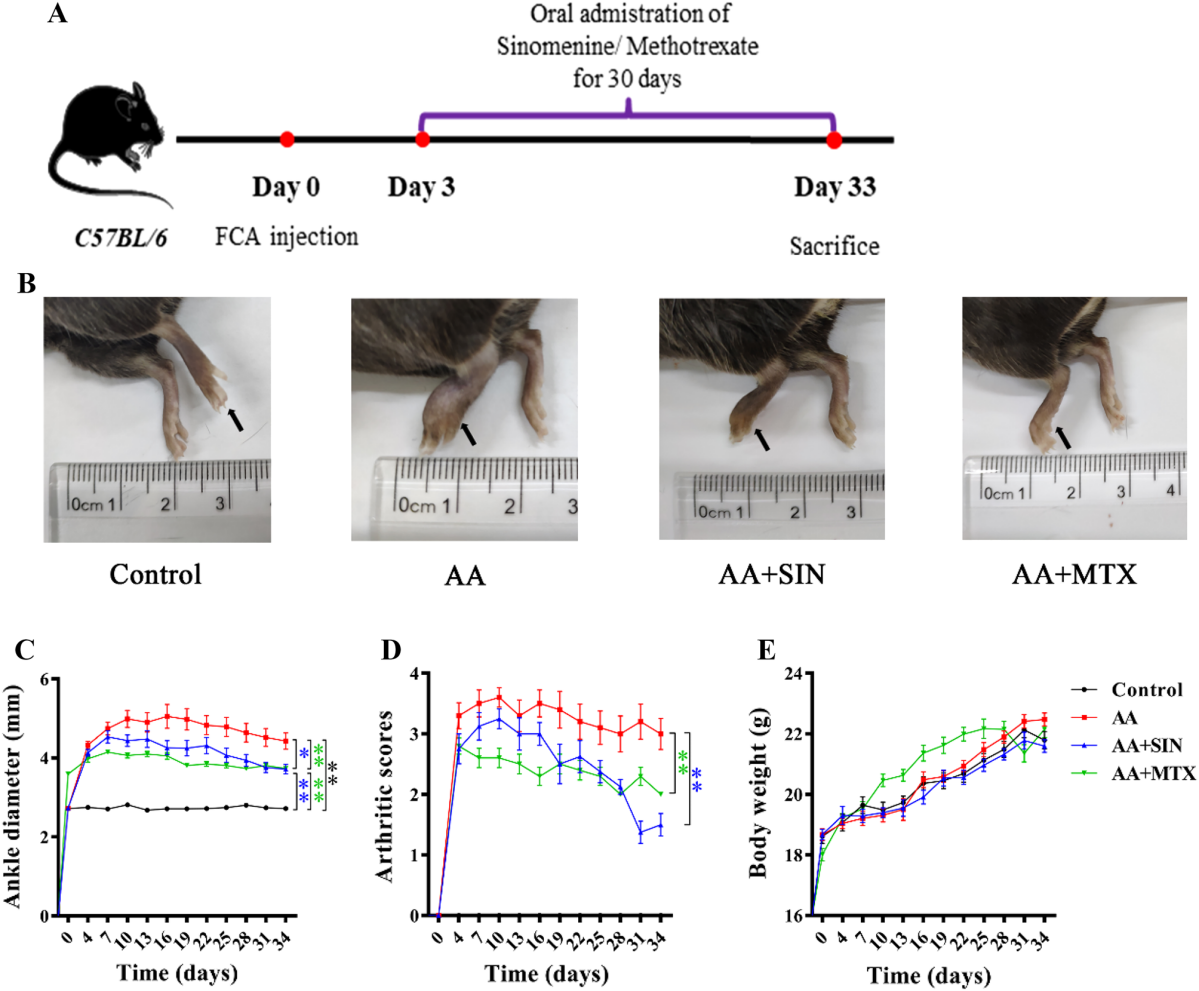
Sinomenine reduced ankle joint swelling and arthritis scores in AA mice. (**A**) Schematic diagram of the animal experiment. (**B**) Representative pictures of the hind paws of the mice on Day 33. The left hind paws are indicated by black arrows. (**C**) The diameter of the ankle joint was measured every three days. The data are shown as the mean ± SD (n = 10). (**D**) The severity of arthritis was scored 0–4. The data are shown as the mean ± SD (n = 10). (**E**) The bodyweight of the mice was monitored. The data are shown as the mean ± SD (n = 10). Control, AA, AA + SIN and AA + MTX indicate the control group, the AA model group, the Sinomenine treatment group and the methotrexate treatment group, respectively. *p < 0.05; **p < 0.01*.*

C57BL/6 mice were randomly grouped according to their body weight. After adaptive feeding for seven days, FCA injection was performed to create an AA model. Three days later, Sinomenine was administered orally at a dose of 90 mg/kg/d, and the treatment of methotrexate was administered orally at a dose of 1.73 mg/kg/3d as the positive control group. For the control and AA groups, an equal volume of saline was administered to the mice in the same way. On the 33rd day, the mice were sacrificed. Serum and joint tissues were collected (Fig. 1A).

During the experiment, the degree of joint swelling was scored. Meanwhile, the joint diameter and weight of the mice were measured every three days. We found that the joints of the mice in the AA group were swollen and that their toes were affected. The joint diameter of the mice in the AA group reached 5 mm on the 10th day, and the joint score reached a maximum of 4. As expected, the swelling was reduced after Sinomenine treatment (Fig. 1B). Compared with the model group, the joint diameter (*p* < 0.05) and joint score (*p* < 0.01) of the mice in the Sinomenine treatment group were significantly reduced (Fig. 1C, D). In addition, Sinomenine had no effect on the weight of the mice (Fig. 1E). Therefore, Sinomenine could significantly reduce the ankle swelling and arthritis score of the AA mice.

### Sinomenine protected bone and cartilage in AA mice

**Figure 2:**
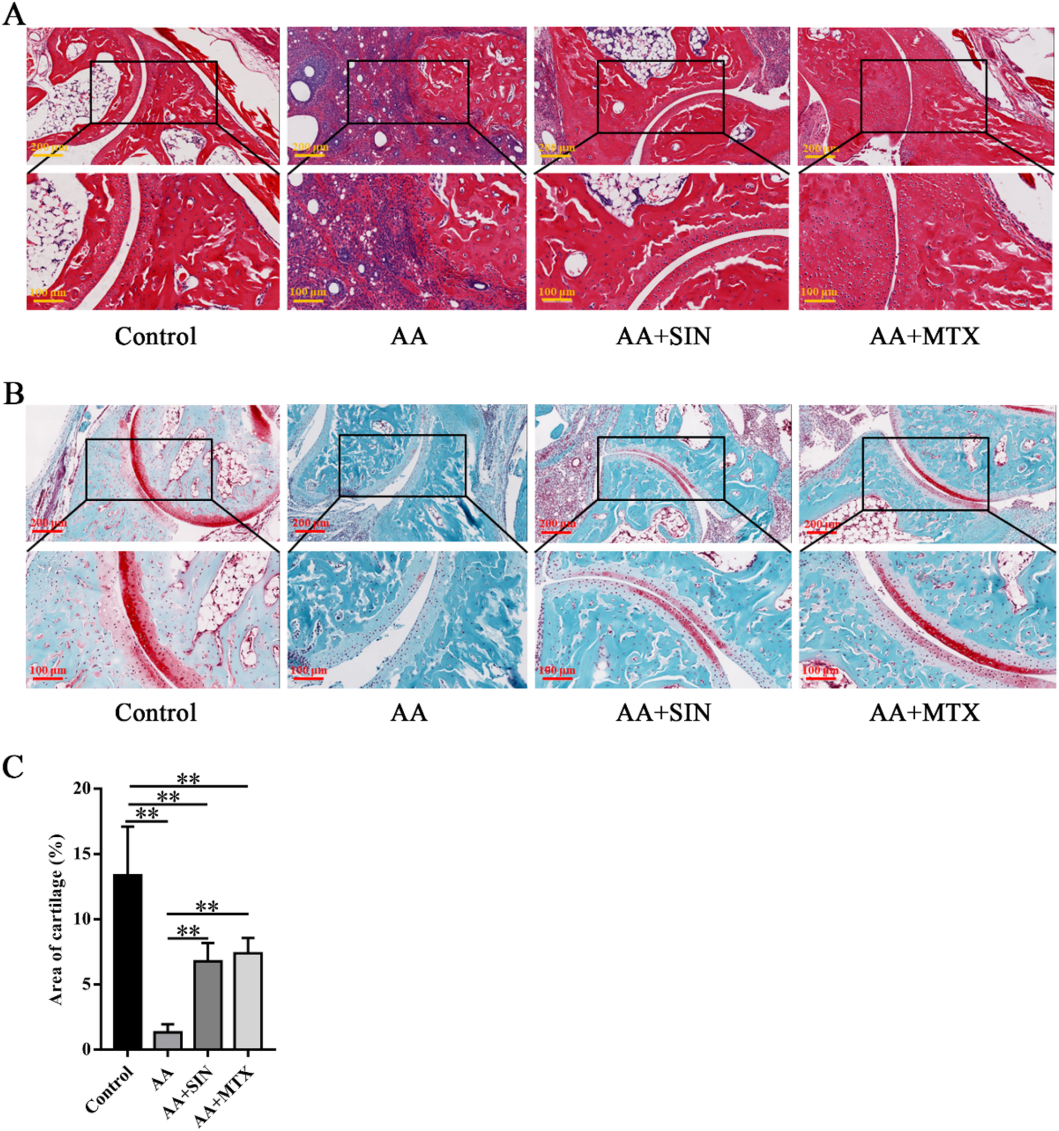
Sinomenine protected bone tissues in AA mice. (**A**) H&E staining of the ankle joint sections from mice in the control group (Control), the model group (AA), the Sinomenine-treated group (AA + SIN) and the methotrexate-treated group (AA + MTX). Representative images are shown. (**B**) Safranin O-fast staining of joint tissue sections from the mice. Representative images are shown. (**C**) Statistical analysis was applied to calculate the cartilage area in (**B**). The data are shown as the mean ± SD (n = 5). *p < 0.05, **p < 0.01*.* “n = 5” represents five mice with representative joint diameters and arthritis scores who were selected from each group to prepare ankle joint tissue sections, and one representative picture from each mouse was used for quantification.

H&E staining was performed to assess the histopathological changes. In the model group, the joints of the mice showed severe synovitis, bone erosion and excessive infiltration of immune cells. In the Sinomenine treatment group, the bone tissues were comparatively intact (Fig. 2A). Then, safranin O-fast staining was performed to assess cartilage damage. In this assay, the redness around the bone indicated cartilage (Fig. 2B). We found that the cartilage of the model group was damaged since its redness declined significantly. Sinomenine intervention helped to alleviate the damage (Fig. 2C). Although Sinomenine failed to block arthritis progression in mice, it succeeded in ameliorating the pathologic changes to some extent.

### Sinomenine reduced systemic and local inflammation in the AA mice

**Figure 3:**
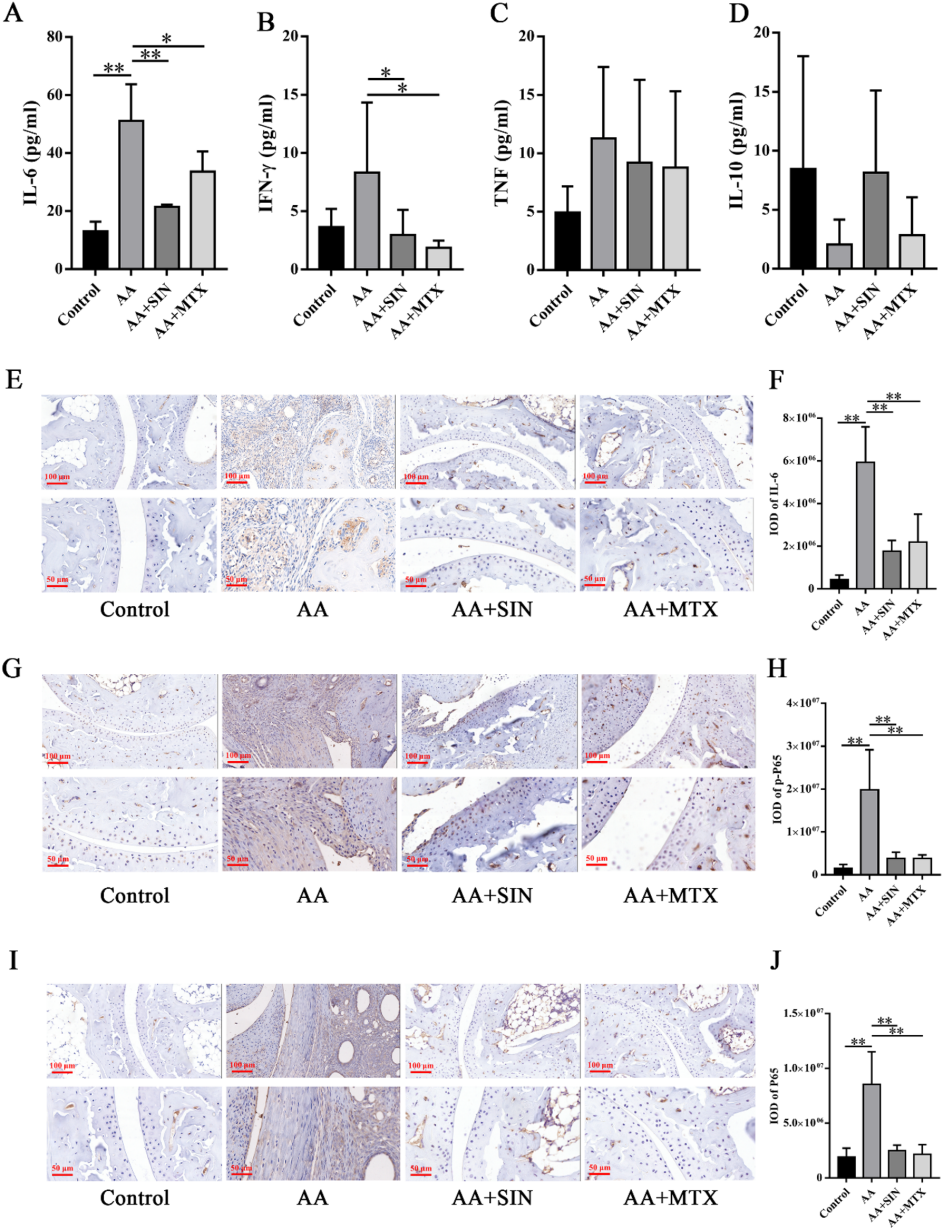
Sinomenine reduced systemic and local inflammation in AA mice. (**A**) The concentration of IL-6 in the serum of mice. (**B**) The concentration of IFN-γ in the serum of mice. (**C**) The concentration of TNF in the serum of mice. (**D**) The concentration of IL-10 in the serum of mice. The data are shown as the mean ± SD (n = 6). (**E**) IHC staining was used to detect IL-6 in ankle joint sections. Representative images are shown. (**F**) Statistical analysis was applied to calculate the IOD of IL-6 in (**E**). The data are shown as the mean ± SD (n = 3). (**G**) IHC staining was used to detect p-P65 in ankle joint sections from the mice. Representative images are shown. (**H**) Statistical analysis was applied to calculate the IOD of p-P65 in (**G**). The data are shown as the mean ± SD (n = 3). (**I**) IHC staining was used to detect P65 in ankle joint sections from the mice. Representative images are shown. (**J**) Statistical analysis was applied to calculate the IOD of P65 in (**I**). The data are shown as the mean ± SD (n = 3). *p < 0.05, **p < 0.01. “n = 3” represents three mice with representative joint diameters; the arthritis scores were selected from each group to prepare ankle joint tissue sections, and one representative picture from each mouse was used for quantification*.*

We used a CBA kit to detect the cytokine concentration in the serum. We found that the concentration of IL-6 in the AA mice was significantly higher than that in the model group. IL-6 and Interferon-γ (IFN-γ) in the Sinomenine treatment group were greatly decreased (Fig. 3A, B), tumor necrosis factor-α (TNF-α) (Fig. 3C) and interleukin-10 (IL-10) (Fig. 3D) showed no differences among the groups.

IL-6 is considered a typical proinflammatory cytokine and it is closely associated with the disease process of RA^35^. Meanwhile, IL-6 blockade has shown great efficacy in treating patients who fail to respond to conventional therapies^36^. Zhou Lan et al. found that Sinomenine downregulated the level of IL-6 in an adjuvant-induced arthritis rat model in a dose-dependent manner^37^. In our study, we found that IL-6 in the serum of mice in the SIN treatment group was decreased significantly. To explore IL-6 expression in the local joints, we performed immunohistochemical assays. The increased IL-6 expression in the AA mice was reduced by Sinomenine (Fig. 3E, F), supporting the previous finding that Sinomenine decreased IL-6 secretion.

NF-κB is the main transcription factor responsible for the expression of IL-6. Phosphorylation of NF-κB can enhance its nuclear localization and transcriptional activity, which promotes the expression of IL-6^38^. In our study, we found that Sinomenine could significantly inhibit the expression of P65 (*p* < 0.05) and p-P65 (*p* < 0.05) in joints (Fig. 3G–J). In summary, Sinomenine may regulate IL-6 via the NF-κB pathways in vivo.

### Sinomenine suppressed LPS-induced signaling pathways in neutrophils in vitro

**Figure 4:**
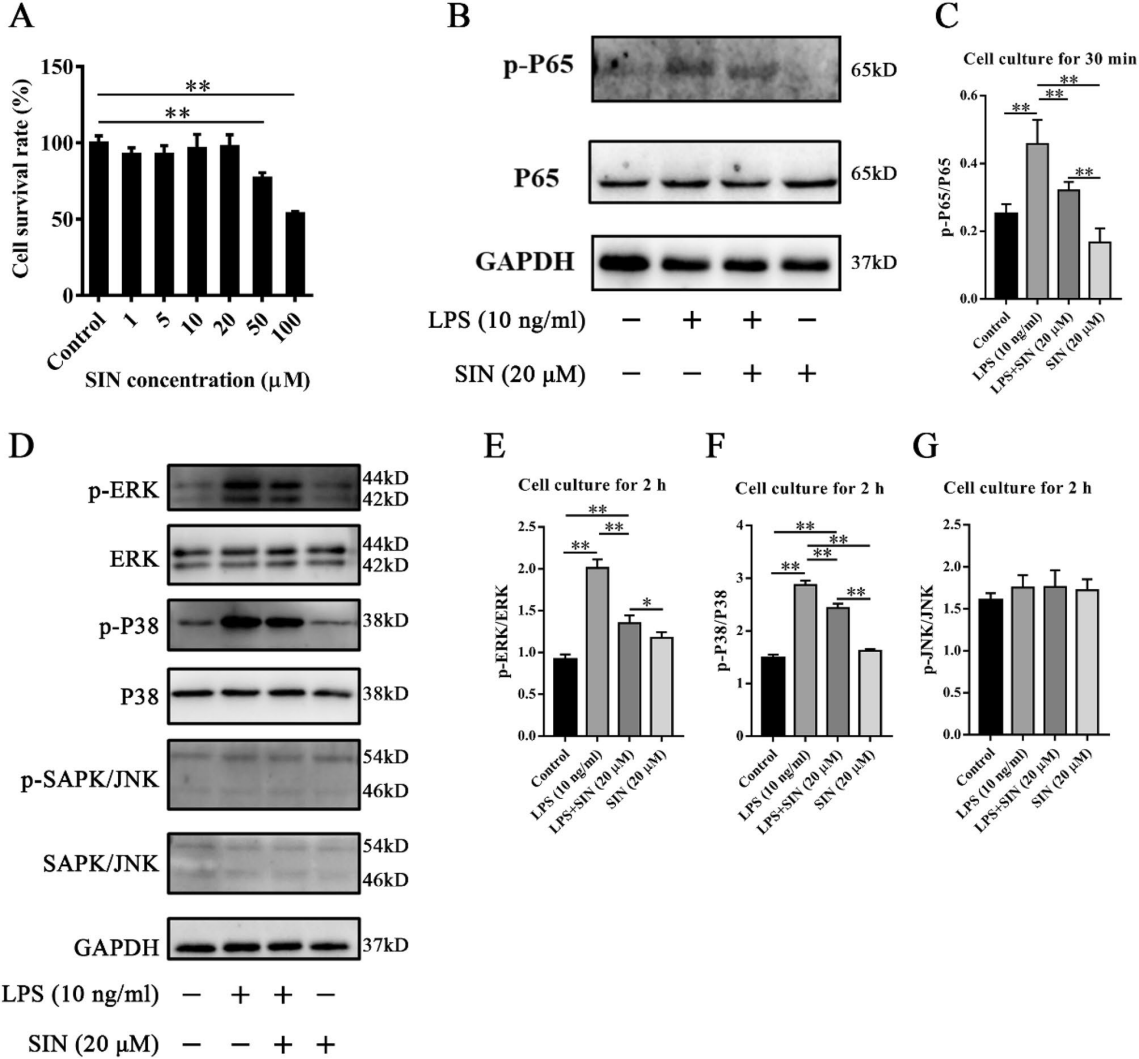
Sinomenine inhibited the inflammatory activity of neutrophils in vitro. (**A**) The effect of Sinomenine on the activity of neutrophils. CCK-8 assays were used to detect the effect of Sinomenine on neutrophil activity after four hours of culture. Statistical analysis was applied to calculate the cell survival rate of each group. The data are expressed as the mean ± SD (n = 6). (**B**) Western blot analysis of the levels of p-P65 and P65 in neutrophils of each group with different treatments. GAPDH was used as an internal control. Representative images are shown (working concentration of p-P65 and P65 antibodies: 1:1000). (**C**) The relative levels of p-P65/P65 in (B). The data are shown as the mean ± SD (n = 3). (**D**) Western blot analysis of the levels of p-ERK, ERK, p-P38, P38, p-JNK and JNK. GAPDH was used as an internal control. Representative images are shown (working concentration of p-ERK, ERK, p-P38, P38, p-JNK and JNK antibodies: 1:1000). (**E**) The relative levels of p-ERK/ERK in (D). The data are shown as the mean ± SD (n = 3). (**F**) The relative levels of p-P38/P38 in (D). The data are shown as the mean ± SD (n = 3). (**G**) The relative levels of p-JNK/JNK in (**D**). The data are shown as the mean ± SD (n = 3). *p < 0.05, **p < 0.01.

LPS was applied to neutrophils to construct a cellular inflammation model to explore how Sinomenine inhibited inflammation. The neutrophils used in the experiments were isolated from the abdominal cavity of mice. First, the CCK-8 assay was used to investigate the impact of Sinomenine on the viability of the neutrophils (Fig. 4A). Compared with the control group, we found that 1–20 μM Sinomenine had no significant effect on the viability of neutrophils, while 50 μM and 100 μM Sinomenine significantly reduced the viability of neutrophils (*p* < 0.01). Western blotting (WB) was used to detect the activation of the NF-κB and MAPK pathways. We found that Sinomenine could significantly inhibit the phosphorylation of p65 at 30 min compared with that in the LPS-treated group (*p* < 0.05) (Fig. 4B, C). After two hours of coculture with LPS, the results showed that Sinomenine could significantly reduce the phosphorylation level of ERK (*p* < 0.01) (Fig. 4D, E) and P38 (*p* < 0.01) (Fig. 4D, E) but had no effect on the expression of p-JNK (Fig. 4D, G). Therefore, our results indicated that Sinomenine regulates LPS-induced inflammation in part through the MAPK signaling pathway and the NF-κB pathway.

### Sinomenine inhibited the migration of neutrophils to local joints

**Figure 5:**
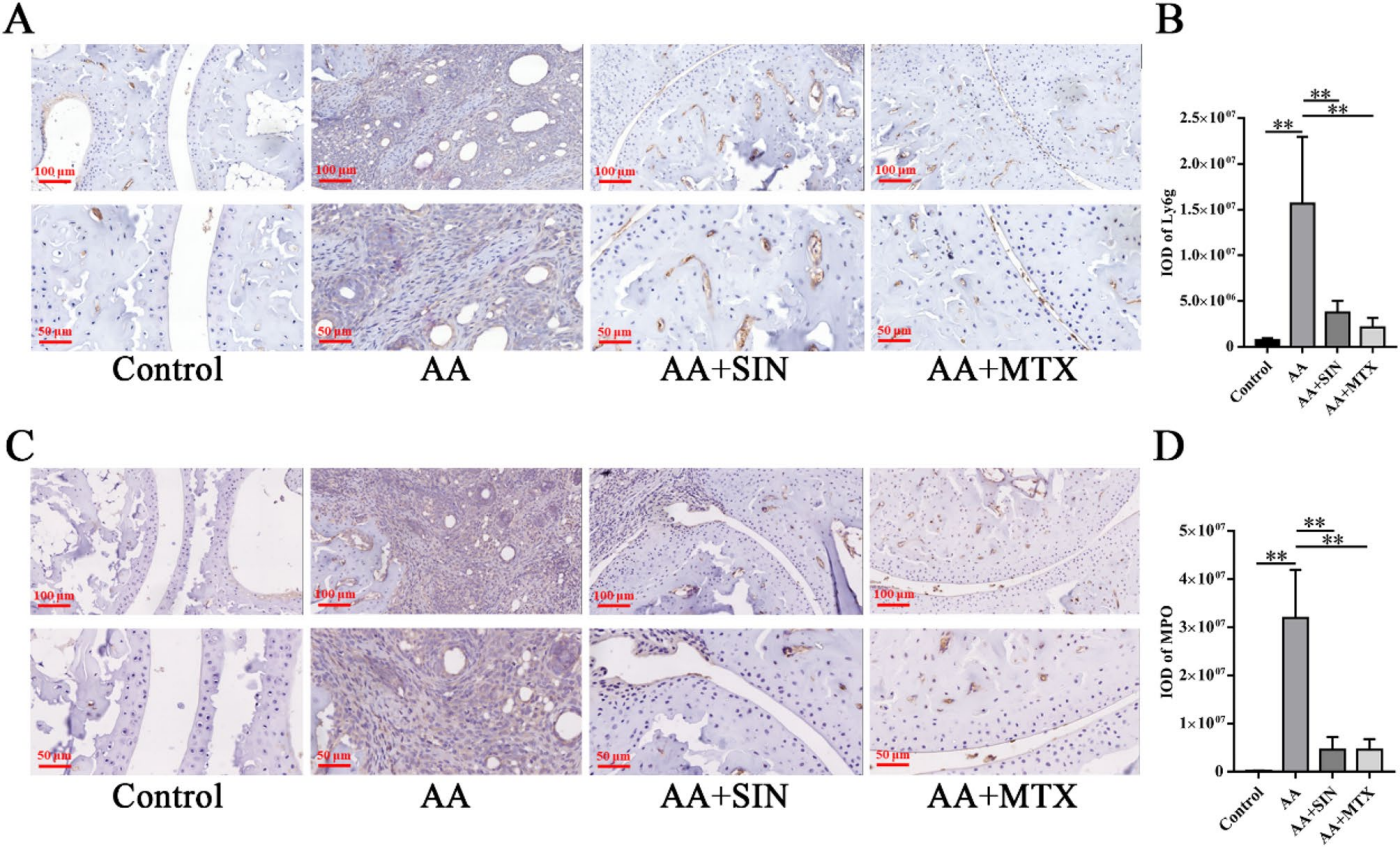
Sinomenine inhibited the migration of neutrophils to local joints. (**A**) IHC assays were performed to measure Ly6G in ankle joint sections. Representative images are shown. (**B**) The IOD of Ly6G in (A). The data are expressed as the mean ± SD (n = 3). (**C**) IHC staining was used to determine the level of MPO in the ankle joint sections from the mice. Representative images are shown. (**D**) The IOD of MPO in (**C**). The data are expressed as the mean ± SD (n = 3). *p < 0.05, **p < 0.01*.* “n = 3” represents three mice with representative joint diameters and arthritis scores that were selected from each group to prepare ankle joint tissue sections, and one representative picture of each mouse was used for quantification.

To investigate the infiltration of neutrophils, immunohistochemistry assays were adopted to detect their characteristic markers. Among neutrophils, Ly6G and MPO are recognized as signs of neutrophil infiltration and tissue inflammation. Therefore, IHC was performed to measure the expression of Ly6G and MPO in joint tissues. The results showed that the expression of Ly6G (Fig. 5A, B) and MPO (Fig. 5C, D) was abundant in the model group but was significantly reduced in the ankles of Sinomenine-treated mice. These results indicated that Sinomenine significantly inhibited the infiltration and activation of neutrophils in AA mice.

### Sinomenine inhibited the formation of NETs in vivo and in vitro

**Figure 6:**
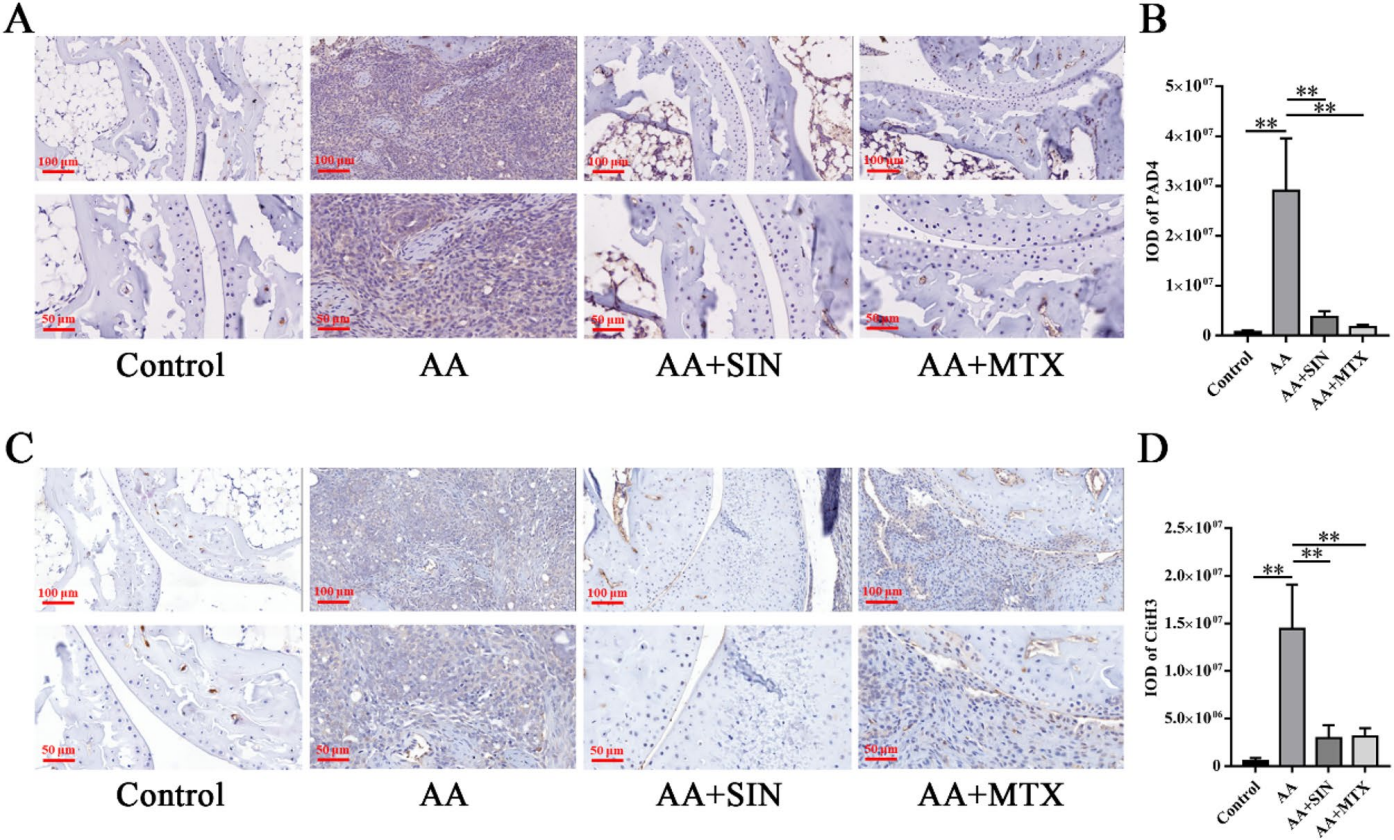
Sinomenine inhibited the formation of NETs in vivo. (**A**) IHC staining was used to determine the level of PAD4 in ankle joint sections from mice. Representative images are shown. (**B**) The IOD of PAD4 in (**A**). The data are shown as the mean ± SD (n = 3). (**C**) IHC staining was used to determine the level of CitH3 in the ankle joint sections from the mice in each group. Representative images are shown. (**D**) The IOD of CitH3 in (C). The data are expressed as the mean ± SD (n = 3). *p < 0.05, **p < 0.01*.* “n = 3” represents three mice with representative joint diameters and arthritis scores that were selected from each group to prepare ankle joint tissue sections, and one representative picture of each mouse was used for quantification.

Specific autoantibodies are believed to aggravate autoimmune responses in RA patients^39–41^. The induction of autoantibodies is partly attributed to NETs. During the formation of NETs, MPO mediates the oxidative activation of NE. PAD4 mediates the production of autoantigens, thus playing an important role in the pathogenesis of RA^42,43^. Anti-PAD4 antibodies can be used as a biomarker of the severity of RA^44^. To determine whether Sinomenine can play a role in inhibiting the production of NETs, we used PAD4 and CitH3 antibodies to perform immunohistochemistry assays. In the model group, PAD4 (Fig. 6A, B) and CitH3 (Fig. 6C, D) were highly expressed in the ankle joints of AA mice, and Sinomenine treatment significantly inhibited the expression of PAD4 (*p* < 0.05) and CitH3 (*p* < 0.01). Therefore, Sinomenine might inhibit NETosis of ankle joints in vivo.

**Figure 7:**
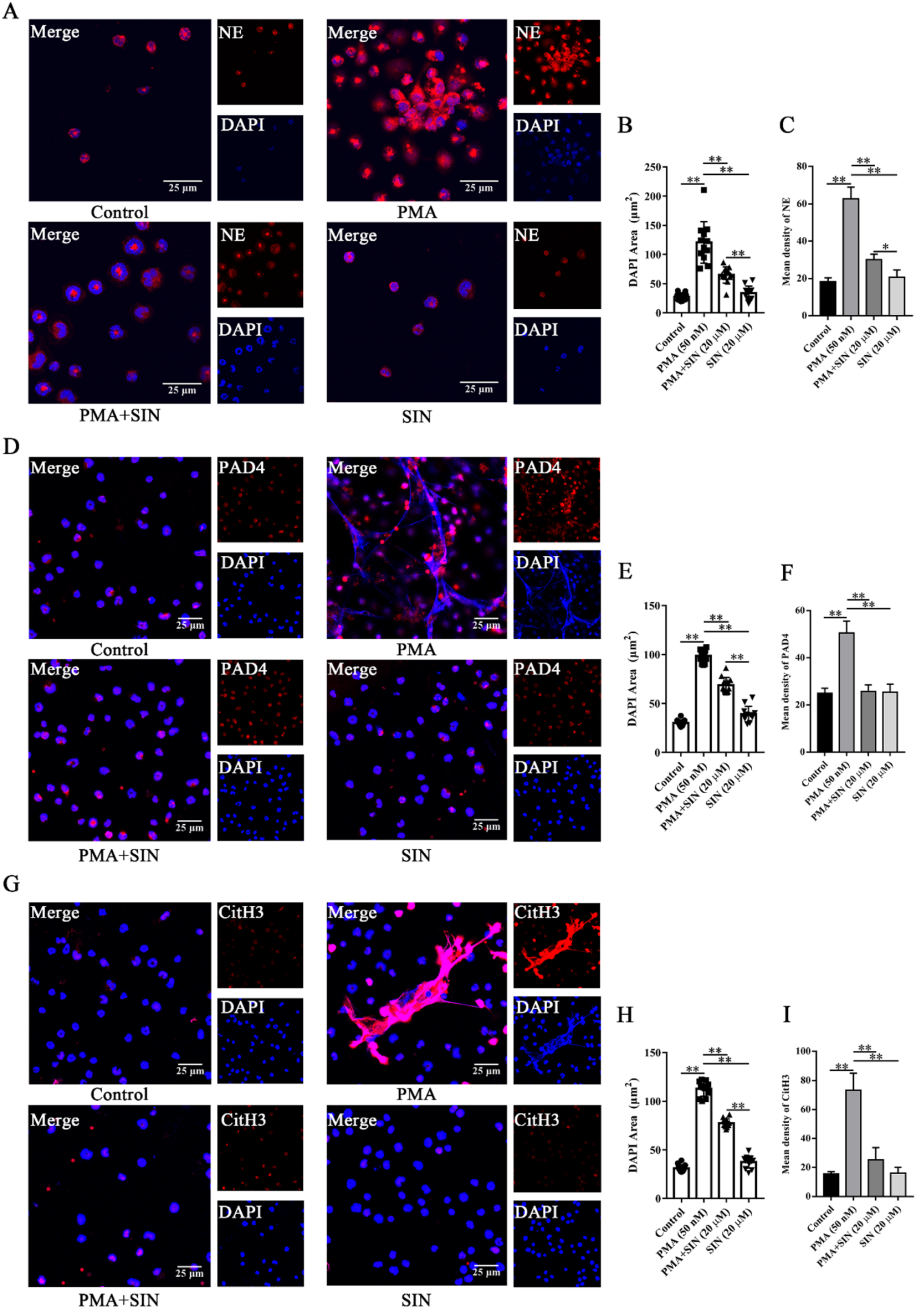
Sinomenine inhibited the formation of NETs in vitro. (**A**) IF staining was used to determine the level of NE in neutrophils. Representative images are shown. (**B**) The nuclear areas in (**A**). The data are shown as the mean ± SD. (**C**) The mean density of NE in (**A**). The data are shown as the mean ± SD (n = 3). (**D**) IF staining was used to determine the level of PAD4 in neutrophils in each group. Representative images are shown. (**E**) The nuclear areas in (**D**). The data are shown as the mean ± SD. (**F**) The mean density of PAD4 in (**D**). The data are shown as the mean ± SD (n = 3). (**G**) IF staining was used to determine the level of CitH3 in the neutrophils in each group. Representative images are shown. (**H**) The nuclear areas in (**G**). The data are shown as the mean ± SD. (**I**) The mean density of CitH3 in (**G**). The data are shown as the mean ± SD (n = 3). *p < 0.05, **p < 0.01*.*

We further explored the effect of Sinomenine on NETs in vitro. The visualization of NETs was accomplished by immunofluorescence assays in which NE, PAD4 and CitH3 were targeted and PMA was added to induce NETosis in vitro*.* Here, the nuclei are marked in blue by DAPI, and NE, PAD4 and CitH3 are marked in red. The PMA-induced NETs diagrams are shown in Fig. 7A, D, G, where the nuclei of neutrophils became larger and the expression of NE, PAD4 and CitH3 increased. However, Sinomenine could significantly inhibit the enlargement of the nucleus and the decondensation of chromatin (Fig. 7B, E, H). The expression of NE, PAD4 and CitH3 also decreased significantly after SIN treatment (Fig. 7C, F, I). Therefore, Sinomenine inhibited the formation of NETs in vitro.

### Sinomenine inhibited PMA-induced autophagy of neutrophils

**Figure 8:**
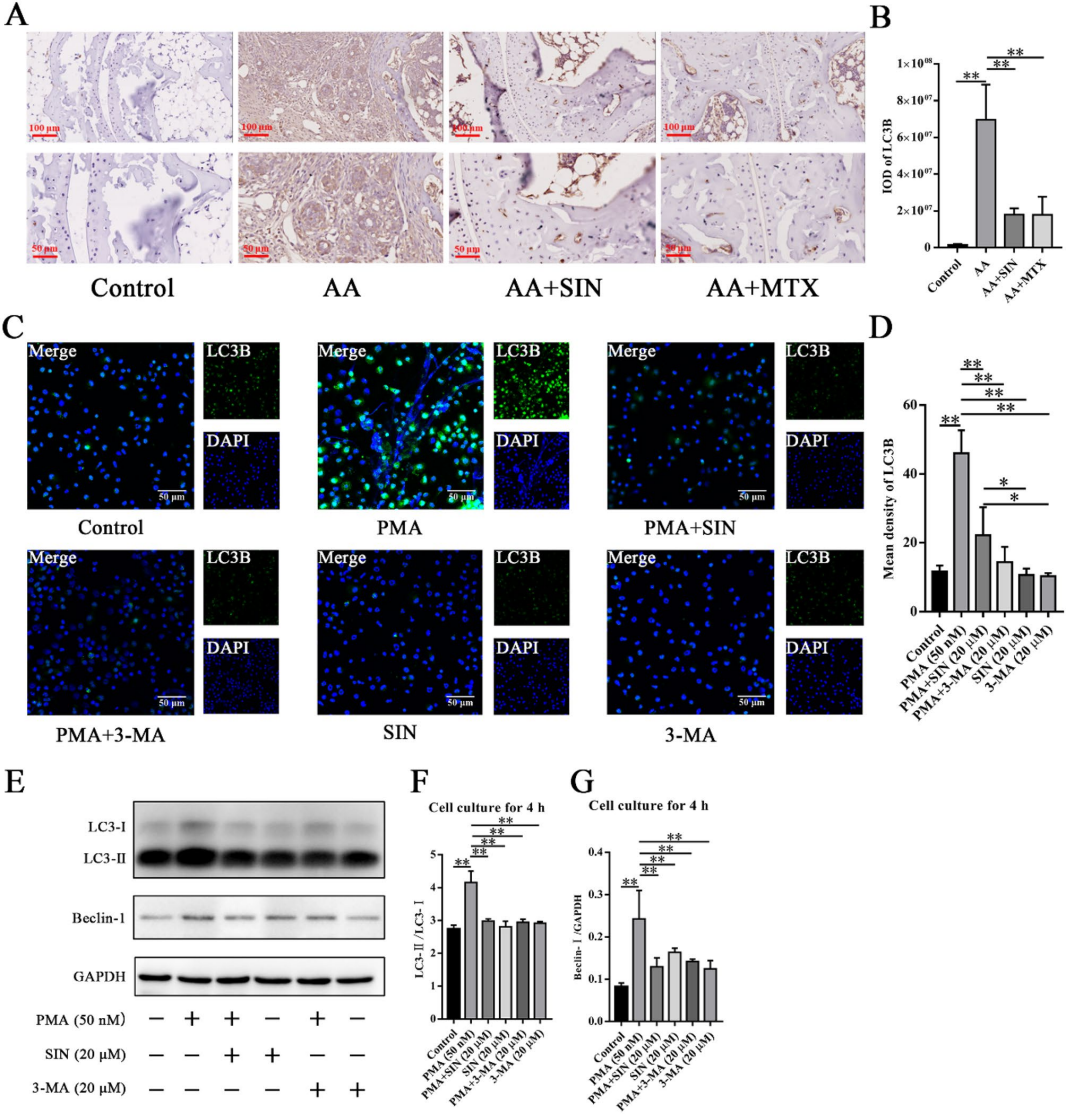
Sinomenine inhibited neutrophil autophagy in vivo and in vitro. (**A**) IHC staining was used to determine the level of LC3B in ankle joint sections from mice. Representative images are shown. (**B**) The IOD of LC3B in (**A**). The data are shown as the mean ± SD (n = 3). “n = 3” represents three mice with representative joint diameters and arthritis scores that were selected from each group to prepare ankle joint tissue sections, and one representative picture of each mouse was used for quantification. (**C**) IF staining was used to determine the level of LC3B in neutrophils from each group. Representative images are shown. (**D**) The mean density of LC3B in (**C**). The data are shown as the mean ± SD (n = 3). (**E**) Western blot analysis of the levels of LC3 and Beclin-I in neutrophils with different treatments for 4 h. Representative images are shown. (**F**) ImageJ was applied to analyze the relative values of LC3-II/LC3-I in (**E**). The data are shown as the mean ± SD (n = 3). (**G**) Statistical analysis was applied to analyze the relative Beclin-I/GAPDH ratios in (**E**). The data are shown as the mean ± SD (n = 3). *p < 0.05, **p < 0.01*.*

Autophagy influences cell survival by various mechanisms, from maintaining cell bioenergetics to clearing protein aggregates and damaged organelles^45^. Recent studies have demonstrated the important roles of neutrophil autophagy and superoxide in NETs formation^40,46^. In the presence of inflammatory factors and microbial pathogenic factors, activated neutrophils can stimulate the autophagy pathway and release NETs. Autophagy can induce the production of ROS and NETs^47^. Yiming Chen et al. found that compared with lymphocytes from healthy subjects, lymphocytes from RA patients showed higher levels of autophagy that were significantly related to inflammation^48^. To determine whether Sinomenine is effective in regulating autophagy, we measured the expression of autophagy-related proteins in vivo and in vitro. IHC staining showed that the expression of LC3B in the ankle joint of AA mice was increased, and Sinomenine could significantly inhibit the expression of LC3B (Fig. 8A, B). For the in vitro studies, IF showed that Sinomenine could significantly inhibit the upregulation of LC3B caused by PMA in neutrophils (Fig. 8C, D). 3-Methyladenine (3-MA) is the specific inhibitor of autophagy. Consistently, WB showed that Sinomenine downregulated the expression of Beclin-1 and suppressed the conversion from microtubule-associated protein 1 light chain 3-I (LC3-I) to LC3-phosphatidylethanolamine family protein conjugation (LC3-II) in PMA-induced neutrophils (Fig. 8E–G). These results showed that Sinomenine significantly inhibited neutrophil autophagy.

## Discussion

RA is an inflammatory autoimmune disease^49^. In RA, the overreaction of the autoimmune system will summon a large number of inflammatory cells, such as neutrophils, T cells, macrophages and fibroblasts, to the joint space, resulting in joint swelling, redness and eventually cartilage and bone damage. In our study, an AA model was applied. The joint diameters and scores of the mice were tested after successful modeling. We found that Sinomenine could significantly inhibit joint swelling and reduce the joint scores of the mice. We performed H&E staining and safranin O-fast staining on the ankle joints to evaluate the local joint pathology. Sinomenine significantly inhibited synovial hyperplasia and cartilage damage in the joints of AA mice. The reduction in bone erosion and cartilage destruction in response to Sinomenine could also be detected by tartrate-resistant acid phosphatase (TRAP) and proteoglycan staining, respectively^50^.

The main feature of RA is an outbreak of inflammation. We found that Sinomenine could suppress the secretion of IL-6. The IHC staining showed that Sinomenine could significantly inhibit the expression of IL-6, p-P65, and P65 in the joints. WB showed Sinomenine downregulated p-P65, p-ERK and p-P38 after LPS exposure in vitro. These results showed that Sinomenine could reduce the inflammatory response of neutrophils in vivo and in vitro*,* where the MAPK and NF-κB signaling pathways participated in the regulation. Similar results have been found in other studies. In the CIA model, Sinomenine has been reported to modulate osteoclast-related cytokines such as RANKL, IL-6, IL-17 and matrix metalloproteinases (MMPs)^30^. Yifan Wu et al. also found that Sinomenine activated the nuclear factor erythroid 2-related factor 2/ heme oxygenase-1 (Nrf2/HO-1) signaling pathway and suppressed the NF-κB signaling pathway to inhibit the inflammatory response and cartilage destruction in osteoarthritis (OA) mice^51^. Xiaoqing Wang et al. used a dorsal root ganglion (DRG) cell line and spinal nerve ligation (SNL) model to study the inhibitory effect of Sinomenine on DRG neuropathic pain and found that Sinomenine had inhibitory effects on neuralgia by reducing dorsal root ganglion inflammation through the p38-MAPK/CREB signaling pathway^52^.

The functions of neutrophils in RA are essential in that they affect all stages, from driving the loss of immune tolerance to joint synovial inflammation. Neutrophils are recruited to inflamed joints and promote additional neutrophil recruitment by the release of chemokines^53^. The recruited neutrophils continuously release cytokines and affect other immune cells. We found that neutrophils were prevented from migrating to the joints by Sinomenine. In other studies, this inhibitory effect was also mentioned. Jun Li et al. found that Sinomenine could significantly inhibit the infiltration of neutrophils into the lung tissue of acute lung injury mice^54^. It is probable that inhibition of migration is one of the mechanisms by which Sinomenine attenuates inflammation.

Neutrophils provide self-antigens through cell death to trigger an immune response^18^. NETosis is an important mechanism of neutrophil programmed cell death, which influences the autoimmune response^55^. NETs assembled on a decondensed chromatin scaffold form a large extracellular network comprised of cytoplasm and granular proteins. NETs can capture, neutralize and kill bacteria, fungi, viruses and parasites and are believed to prevent the spread of bacteria and fungi^40^. In recent years, researchers have proven that NETs play a significant role in the pathogenesis of RA^56^. Abnormal NETosis is one of the pathogeneses observed in many autoimmune diseases, such as RA and systemic lupus erythematosus (SLE)^57^. NETs are an important source of autoantigens that stimulate ACPAs. ACPAs promote the release of PAD4 from neutrophils. PAD4-mediated histone citrullination and chromatin decondensation are critical to the formation of NETs^57^. The conditions of RA are positively connected to the expression level of PAD4^58^. In our research, the IHC results showed that the expression of PAD4 and CitH3 was inhibited, suggesting that the formation of NETs was decreased by Sinomenine in vivo. The IF results showed that Sinomenine significantly inhibited PMA-induced expression of NE, PAD4 and CitH3 in vitro, as well as the nuclear depolymerization of neutrophils. We propose that the reduction in NETs induced by Sinomenine is important for the alleviation of RA.

Autophagy is a highly conserved process in eukaryotic cells where various things can be selectively degraded by transferring them from the cytoplasm to lysosomes, thus playing a key role in cell homeostasis^59^. LC3B is an autophagy marker related to the formation and maturation of autophagosomes^39^. The transformation of LC3-I into LC3-II promotes the formation of autophagosomes^60^. Beclin-I is a key protein that induces autophagy^61^. Autophagy is closely related to the disease process of RA. It was revealed that the level of autophagy was increased in the neutrophils from RA patients^62^. Yu Wang et al. found that inhibition of autophagy could delay the progression of RA^63^. Besides, a series of studies have proposed that the formation of NETs is closely correlated with autophagy^41,64^. Inhibition of autophagy can prevent intracellular chromatin depolymerization, which is essential for the formation of NETs^46^. Inhibition of autophagy can prevent chromatin degradation and inhibit nuclear depolymerization. We used IHC, IF and WB methods to detect the effect of Sinomenine on autophagy in vivo and in vitro. IHC stained sections showed that Sinomenine significantly reduced the increased expression of LC3B in the ankle joints of AA mice. IF assays showed that Sinomenine significantly inhibited PMA-induced LC3B expression and inhibited the production of autophagosomes. WB results indicated that Sinomenine could significantly inhibit the expression of the autophagy-related proteins LC3-II and Beclin-I. Therefore, our results showed that Sinomenine could inhibit PMA-induced neutrophil autophagy and alleviate RA.

## Conclusion

In conclusion, we demonstrated that Sinomenine could alleviate the symptoms of AA mice by regulating neutrophil activities. Furthermore, the inhibition effect of Sinomenine on NETs formation is closely related to its anti-autophagy effect. Thus, Sinomenine could provide a treatment strategy for RA by targeting neutrophils.

## Supplementary Information

Supplementary Information.

## Data Availability

Anonymized data not published within the article will be shared by request with Guangrui Huang (hgr@bucm.edu.cn) or Kai Yuan (yuankai@bucm.edu.cn).

## Abbreviations

RA: Rheumatoid arthritis
NETs: Neutrophil extracellular traps
CRP: C-reactive protein
ACPAs: Antibodies to citrullinated protein antigens
MPO: Myeloperoxidase
NE: Neutrophil elastase
PAD4: Protein-arginine deiminase type 4
Ly6G: Lymphocyte antigen 6 complex, locus G
CitH3: Citrullinated histone H3
LC3B: Microtubule-associated protein 1 light chain 3B
TRAP: Tartrate-resistant acid phosphatase
MMPs: Matrix metalloproteinases
IL-6: Interleukin-6
IFN-γ: Interferon-γ
TNF-α: Tumor necrosis factor-α
IL-10: Interleukin-10
GAPDH: Glyceraldehyde-3-phosphate dehydrogenase
NF-κB: Nuclear factor kappa-B
MAPK: Mitogen-activated protein kinase
IgG: Immunoglobulin G
MTX: Methotrexate
LEF: Leflunomide
SIN: Sinomenine
RANKL: Receptor activator of NF-κB ligand
OPN: Osteopontin
CIA: Collagen-induced arthritis
HIF-1α: Hypoxia-inducible factor-1α
VEGF: Vascular endothelial growth factor
ANG-1: Angiopoietin 1
AA: Adjuvant-induced arthritis
Nrf2/HO-1: Nuclear factor erythroid 2-related factor 2/heme oxygenase-1
OA: Osteoarthritis
DRG: Dorsal root ganglion
SNL: Spinal nerve ligation
SLE: Systemic lupus erythematosus
FCA: Freund’s complete adjuvant
LPS: Lipopolysaccharide
PMA: Phorbol 12-myristate 13-acetate
CBA: Cytometric beads array
TBST: Tris-buffered saline tween-20
CCK-8: Cell counting kit 8
H&E staining: Hematoxylin and eosin staining
IHC staining: Immunohistochemistry staining
DAB: 3,3'-Diaminobenzidine
SDS–PAGE: Sodium dodecyl sulfate–polyacrylamide gel electrophoresis
PVDF: Polyvinylidene fluoride
BSA: Bovine serum albumin
HRP: Horseradish Peroxidase
3-MA: 3-Methyladenine
SD: Standard deviation
IFN: Interferon
