# Supplementary material for: Sinomenine ameliorates adjuvant-induced arthritis by inhibiting the autophagy/NETosis/inflammation axis
Source: Sci Rep. 2023 Mar 9;13:3933. doi: 10.1038/s41598-023-30922-3 (PMC9998614; doi:10.1038/s41598-023-30922-3)
Supplement: Supplementary file 1 — Supplementary Information. [file 41598_2023_30922_MOESM1_ESM.pdf]

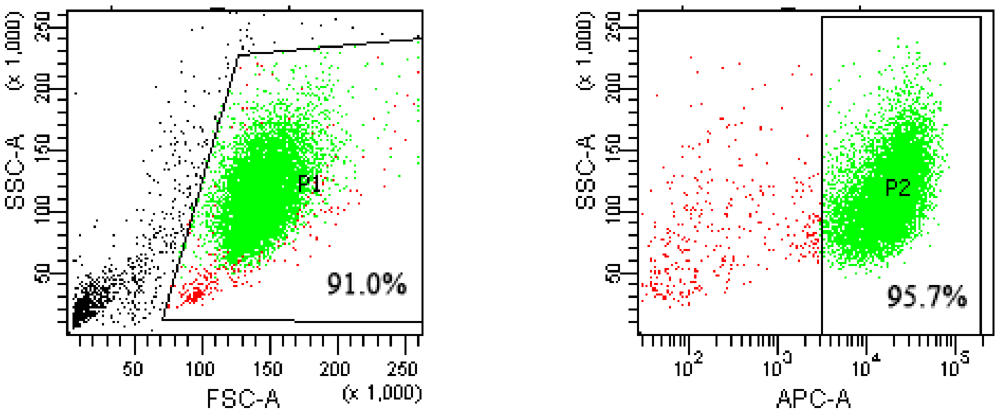

Supplement Figure 1. The purity of neutrophils isolated from the abdominal cavity of mice was tested by flow cytometry.

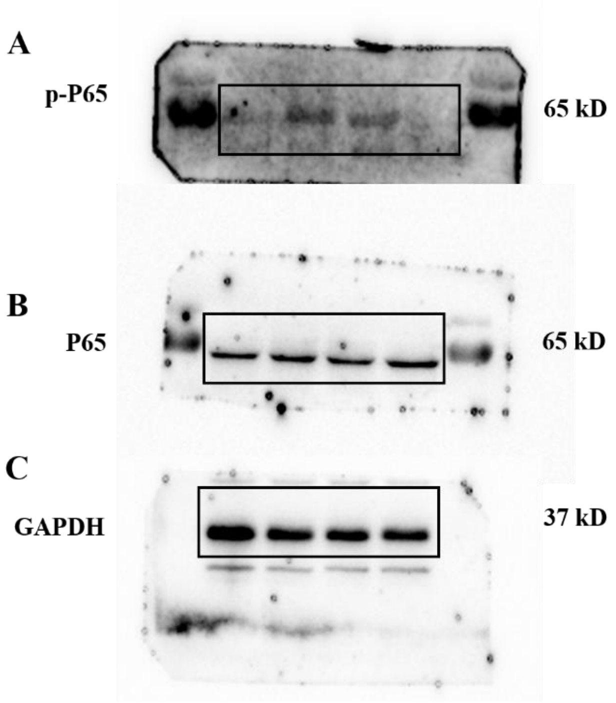

Supplement Figure 2. The complete blotting of Figure 4B. (A) The complete blotting of p-P65. (B) The complete blotting of P65. (C) The complete blotting of GAPDH.

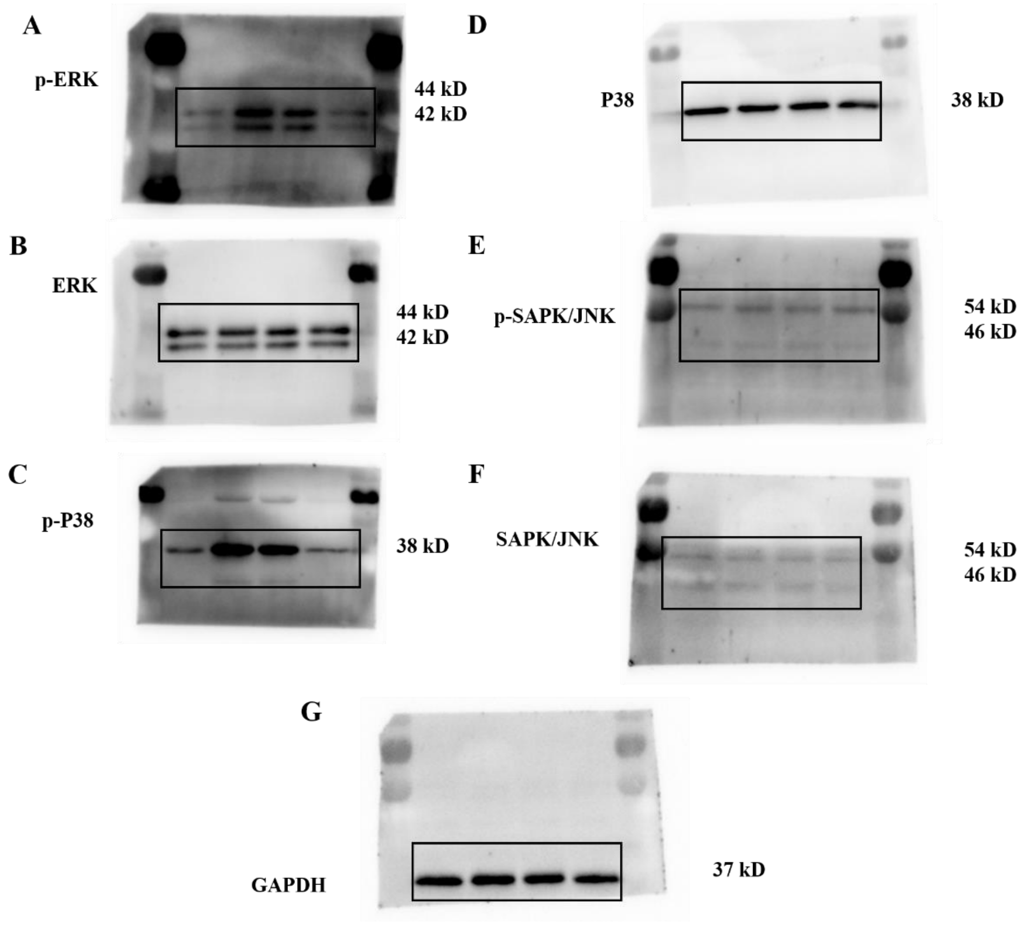

Supplement Figure 3. The complete blotting of Figure 4D. (A) The complete blotting of p-ERK. (B) The complete blotting of ERK. (C) The complete blotting of p-P38. (D) The complete blotting of P38. (E) The complete blotting of p-SAPK/JNK. (F) The complete blotting of SAPK/JNK. (G) The complete blotting of GAPDH.

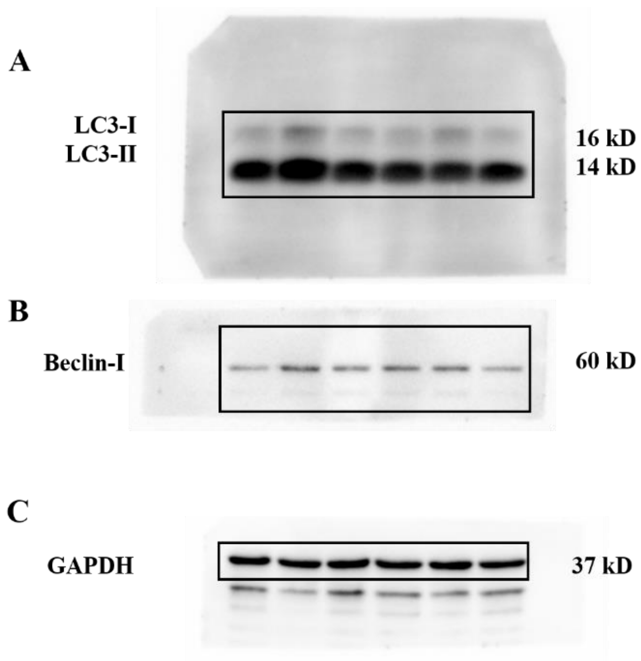

Supplement Figure 4. The complete blotting of Figure 8E. (A) The complete blotting of LC3-II and LC3-I. (B) The complete blotting of Beclin-I. (C) The complete blotting of GAPDH.
